# Vascular etiologies of unilateral pulmonary fibrosis: Case series and literature overview

**DOI:** 10.1016/j.radcr.2025.11.010

**Published:** 2025-12-12

**Authors:** Diletta Cozzi, Luca Gozzi, Edoardo Cavigli, Chiara Moroni, Alessandra Bindi, Elisabetta Rosi, Sara Tomassetti, Vittorio Miele

**Affiliations:** a Department of Emergency Radiology, Careggi University Hospital, Florence, Italy; b Department of Experimental and Clinical Biomedical Sciences “Mario Serio”, University of Florence, Florence, Italy; c Department of Pulmonology, Careggi University Hospital, Florence, Italy; d Unit of Interventional Pulmonology, Department of Experimental and Clinical Medicine, Careggi University Hospital, Florence, Italy

**Keywords:** Lung fibrosis, Unilateral lung fibrosis, HRCT, Pulmonary embolism, CTEPH

## Abstract

Unilateral pulmonary fibrosis (UPF) is an uncommon manifestation of interstitial lung disease that may arise from various causes. Among those, cardiovascular etiology is often overlooked because affected patients do not present history of exposure of any kind, are frequently asymptomatic or present only mild, subtle and nonspecific symptoms. As a result, these cases are usually identified incidentally on imaging performed for unrelated reasons, contributing to under recognition of their true prevalence. We report 3 patients with incidentally detected UPF on high-resolution computed tomography (HRCT), each associated with a distinct vascular abnormality, both congenital and acquired conditions: unilateral absence of the pulmonary artery, chronic pulmonary thromboembolism, and pulmonary artery branch hypoplasia. Our aim with this case series is to highlights the diverse radiologic patterns of vascular UPF, underscoring the importance of considering vascular causes in the differential diagnosis of unilateral fibrotic lung disease.

## Introduction

Pulmonary fibrosis (PF) is typically a bilateral and progressive interstitial lung disease (ILD), often idiopathic, although it can also be secondary to environmental exposure, connective tissue disease or drug toxicity [[1], [2], [3]]. Despite both lungs being exposed to the same toxics or triggers, some degree of asymmetric lung involvement may occur. However, purely or predominantly unilateral pulmonary fibrosis (UPF) is a very rare condition, which may result from specific circumstances such as radiation pneumonitis, chemotherapy, ventilation-induced lung injury, gastroesophageal reflux (GERD) or cardiovascular disorders [[4], [5], [6], [7], [8], [9]]. The latter in particular, are often underrecognized because patients lack a suggestive clinical history prompting targeted imaging or pulmonary function testing [[10], [11], [12]]. Based on etiology, the most relevant vascular causes of UPF can be divided into 2 groups: congenital and acquired conditions. Among congenital causes, unilateral absence of pulmonary artery (UAPA) is the most significant, whereas chronic pulmonary thromboembolism (CPTE) is considered the most common acquired condition, followed by malignancies as slow-growing pulmonary artery sarcoma [[13], [14], [15], [16], [17], [18], [19]]. Another vascular condition that may be responsible for UPF is hypoplasia of a pulmonary artery branch, considered to be both congenital - anatomical variant causing vascular compression - or acquired, as in fibrosing mediastinitis [4,20].

The clinical gap lies in the limited awareness of vascular mechanisms underlying UPF and the corresponding educational need to recognize characteristic high-resolution computed tomography (HRCT) findings that can guide appropriate etiologic work-up.

This paper presents 3 illustrative cases of UPF secondary to distinct vascular pathologies, emphasizing their imaging features and the diagnostic clues that may facilitate earlier recognition of these uncommon entities.

## Case 1

### Clinical presentation

A 58-year-old woman presented after a recent bronchial infection, reporting mild exertional dyspnea (grade ¼ on Modified British Medical Research Council Questionnaire—mMRC) and occasional cough. On examination, fine velcro-like crackles were heard at the right lung base. Pulmonary function tests (PFTs) were normal (FVC 99% [2.88 L], FEV₁ pre-BD 99% [2.30 L], TLC 98%, DLCO 87%), and autoimmune screening was negative. The patient was non-smoker, without family history of ILD, no relevant occupational or environmental exposures. Cardiologic evaluation, including ECG and echocardiography, showed no significant abnormalities.

### Imaging findings

**Fig. 1:**
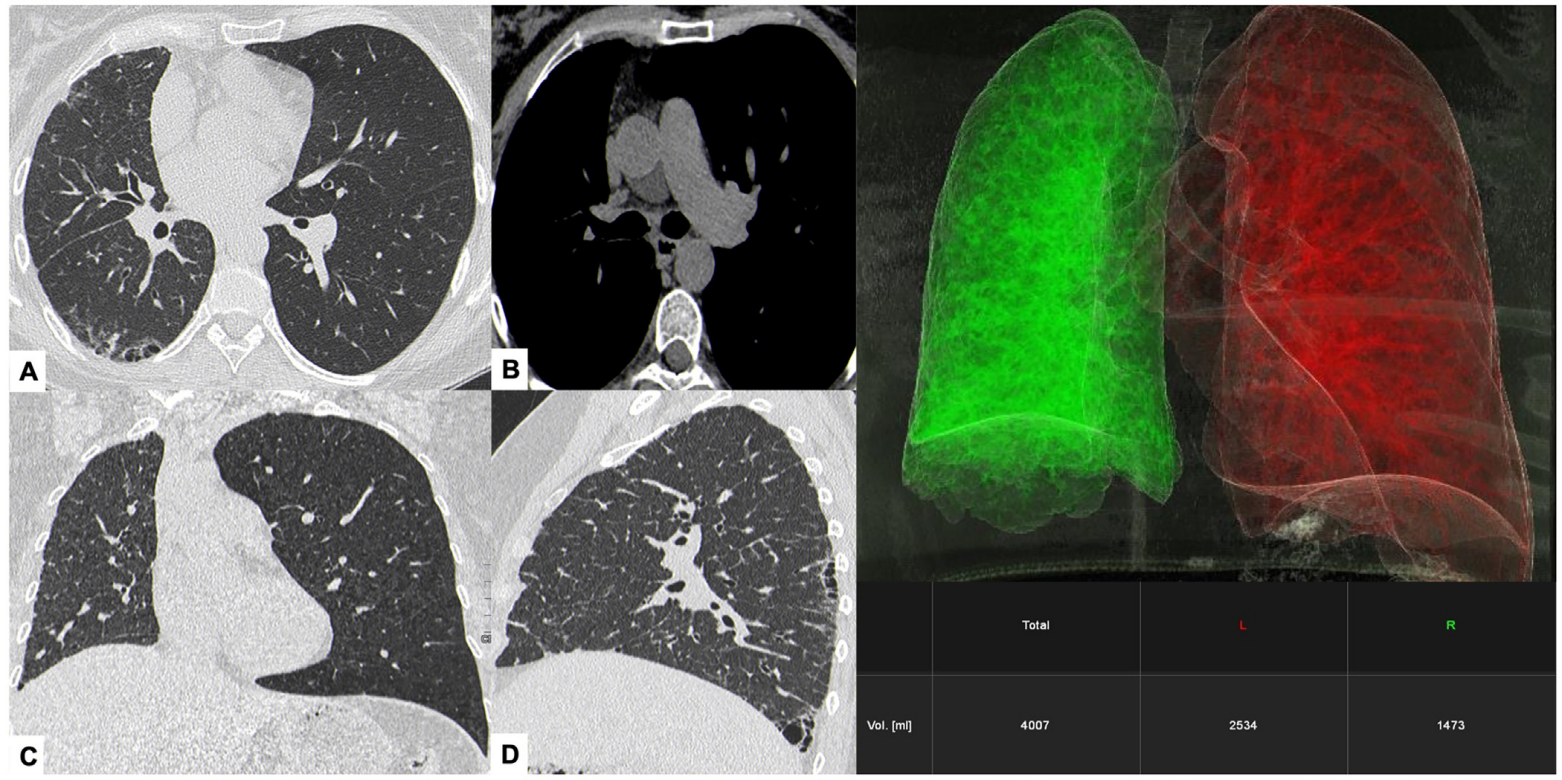
*Case 1 – Unilateral absence of the right pulmonary artery (UAPA) causing unilateral pulmonary fibrosis.* HRCT images show irregular subpleural interstitial thickening and honeycombing-like foci confined to the right lung (white arrows in A, B), resulting in marked asymmetry of lung inflation (C). Mediastinal window reconstruction demonstrates complete agenesis of the right pulmonary artery (arrowhead in D), identified as the underlying cause of the unilateral fibrotic changes. The 3D volumetric reconstruction (right panel) generated using Siemens Healthcare software (syngo.via Pulmo3D) confirms reduced right lung volume compared to the left (1473 mL vs. 2534 mL).

HRCT demonstrated right lung volume loss with irregular interstitial reticulations, traction bronchiolectasis, and subtle honeycombing in a peripheral, basal-predominant distribution—consistent with a usual interstitial pneumonia (UIP)—like pattern confined to the right lung. Evaluation of mediastinal windows revealed complete absence of the right pulmonary artery, suggesting a congenital vascular etiology for the fibrosis (Fig. 1).

### Management and outcome

A multidisciplinary discussion recommended conservative management with clinical and functional monitoring. At 12 - month follow-up, HRCT and PFTs showed no disease progression; therefore, antifibrotic therapy was not initiated.

## Case 2

### Clinical presentation

A 70-year-old man was evaluated during a multidisciplinary meeting for suspected interstitial lung disease (ILD). He had a history of pulmonary embolism (2016), right carotid endarterectomy and atrial flutter (2018), and prior occupational exposure to toxic substances. Echocardiography (2017) demonstrated right atrial enlargement (20 cm²), mild right ventricular dilation with with tricuspid annular plane systolic excursion (TAPSE) at the lower normal limit (15 mm), and an enlarged main pulmonary artery (30 mm). Right heart catheterization confirmed precapillary pulmonary hypertension, and a ventilation/perfusion (V/Q) scan established the diagnosis of chronic thromboembolic pulmonary hypertension (CTEPH). PFTs revealed preserved ventilatory parameters (FEV₁/FVC 87%, FEV₁ 113%, FVC 119%, TLC 99%) with mildly reduced diffusing capacity (DLCO 67%).

### Imaging findings

**Fig. 2:**
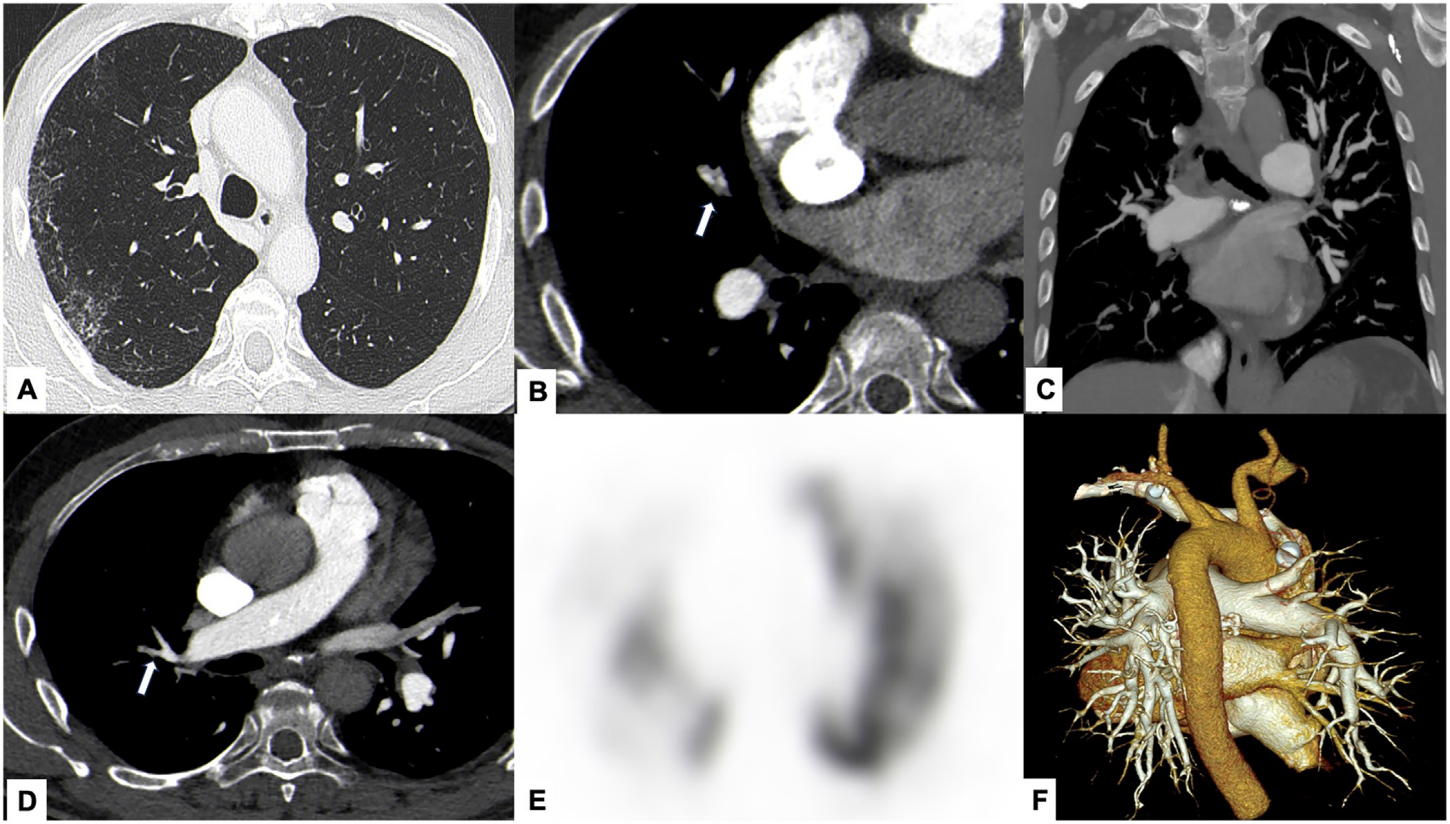
*Case 2 – Chronic pulmonary thromboembolism (CPTE) with secondary unilateral pulmonary fibrosis.* CT pulmonary angiography demonstrates narrowing of right upper lobe pulmonary arteries with eccentric thrombotic material and endoluminal webs (arrows in A, B), consistent with CPTE. Mild unilateral interstitial thickening is visible in the dorsal portion of the right upper lobe (arrows in C). MIP reformatting (D) and VRT 3D reconstruction (E) highlight decreased vascular branching in the affected region. Pulmonary scintigraphy (F) confirms reduced perfusion of the right lung, supporting the diagnosis of chronic right CPTE.

CT pulmonary angiography (CTPA) showed endoluminal webs and eccentric stenoses of the right pulmonary arteries, predominantly in the upper and lower lobes, consistent with chronic thromboembolic changes. Parenchymal assessment demonstrated unilateral interstitial involvement in the right upper lobe with traction bronchiolectasis and subpleural distortion of the major fissure (Fig. 2).

### Management and outcome

PFTs and HRCT findings remained stable over the subsequent 5 years. However, in early 2023 a pancreatic head tumor was discovered, and in late 2024, the patient was admitted to our ED with dyspnea due to a new PE in the right lower lobe. The interstitial findings remained unchanged. The patient later died from metastatic pancreatic cancer.

## Case 3

### Clinical presentation

A 69-year-old man with a history of prostate cancer underwent FDG-PET/CT for post-surgical restaging. The study incidentally revealed hypoinflation of the left lung and an anomalous origin of the left pulmonary artery arising from the right pulmonary artery and coursing retrotracheally, consistent with a pulmonary artery sling.

### Imaging findings

**Fig. 3:**
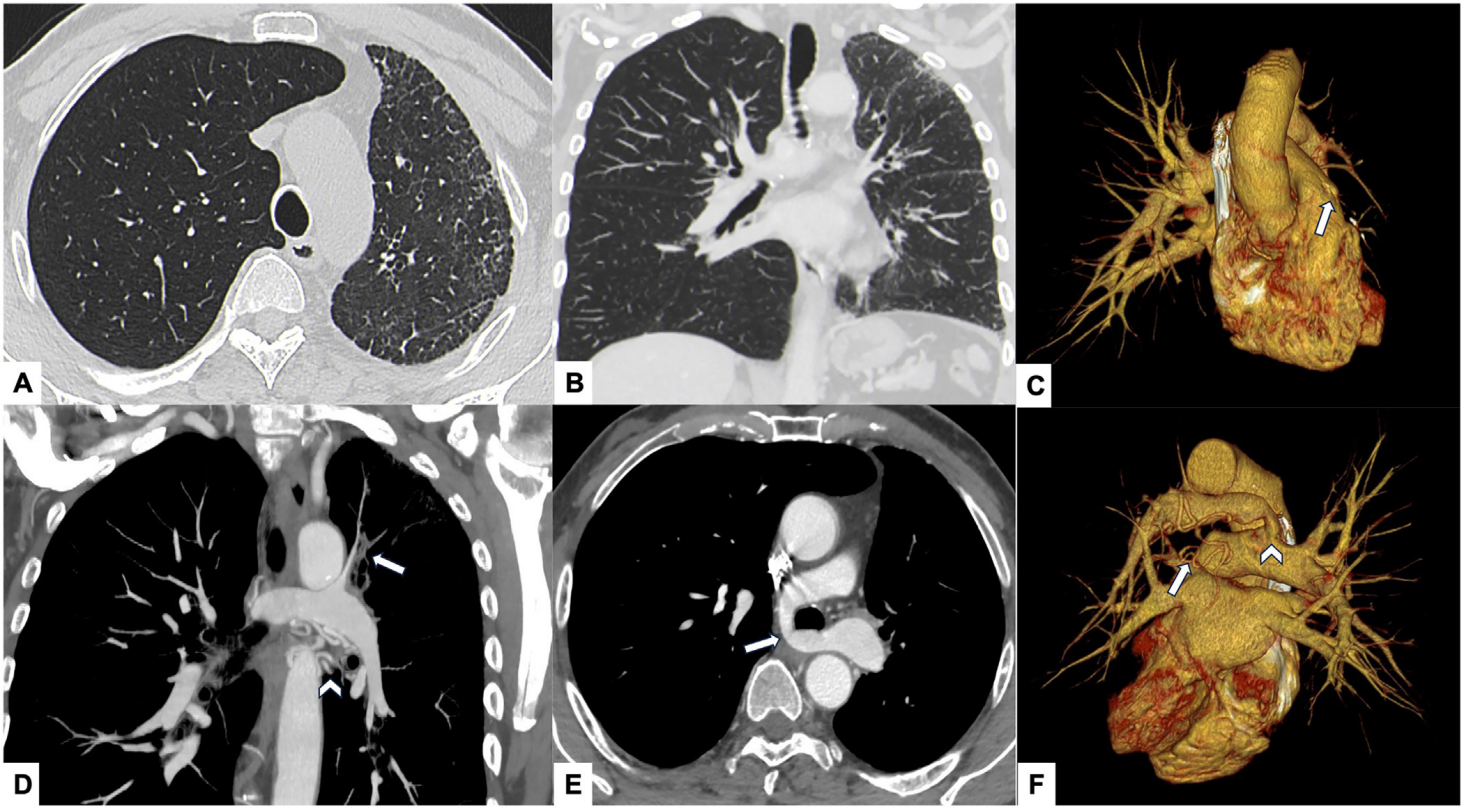
*Case 3 – Pulmonary artery sling with hypoplasia of left pulmonary arterial branches and unilateral fibrotic remodeling.* Axial CT images show hypoinflation of the left lung with fine subpleural interstitial thickening (A, B). After contrast administration, an aberrant left pulmonary artery originating from the right pulmonary artery and coursing retrotracheally is demonstrated (arrow in C), consistent with a pulmonary artery sling. Hypoplasia of several left pulmonary arterial branches (arrow in D) along with hypertrophy of bronchial collateral arteries (arrowhead in D) are also evident. VRT 3D reconstruction (E, F) confirms the absence of the left pulmonary artery in its expected anatomic position (arrow in E and F) and highlights the compensatory systemic collateral circulation (arrowhead in E).

Further CT evaluation showed hypoplasia of multiple segmental and subsegmental branches of the left pulmonary artery supplying both lobes, accompanied by fine subpleural interstitial thickening and traction bronchiectasis (Fig. 3). Prominent hypertrophy of systemic collateral vessels, including the left bronchial and internal mammary arteries, was also observed, reflecting compensatory systemic perfusion secondary to reduced pulmonary arterial flow.

### Management and outcome

No intervention was required, and the findings were attributed to a congenital vascular anomaly causing unilateral fibrotic remodeling of the left lung. The patient remained asymptomatic on follow-up.

## Discussion and conclusion

In this paper, we describe 3 cases of UPF caused by distinct vascular conditions: unilateral pulmonary agenesis, chronic pulmonary thromboembolism, and hypoplasia of left pulmonary artery branches. As previously mentioned, vascular etiologies responsible for UPF are frequently underrecognized due to their indolent course and the lack of specific signs and symptoms prompting timely medical investigation. Recently, several publications have addressed this topic. Two, in particular, have explored unilateral absence of the pulmonary artery (UAPA) examining large patient cohorts and presenting meta-analyses. The first study, by Marrocchio et al., collected 13 cases (2004-2020) of unilateral lung hypoperfusion and UPF: 5 patients (38%) had UAPA, and the remaining eight (62%) had extrinsic pulmonary artery compression secondary to fibrosing mediastinitis (4). In contrast, Wang et al. conducted a comprehensive review of all reported cases of isolated unilateral absence of the pulmonary artery (UAPA) published between 1990 and 2016, identifying UPF in 6 patients (14%) [11]. Despite the mechanisms linking vascular anomalies and pulmonary fibrosis are not fully understood yet, chronic hypoperfusion, oxidative stress, and low-grade inflammation are considered central drivers. Experimental models have shown that prolonged arterial occlusion triggers vascular remodeling, epithelial injury and fibroblast activation. In regions of reduced perfusion, the lung may react with bronchoconstriction, resulting in hypocapnia, reduced alveolar development, impaired mucociliary clearance and ultimately chronic inflammation. Growth factors such as TGF-β, reactive oxygen species (ROS) and cytokines involved in vascular remodeling contribute to fibroblast proliferation and extracellular matrix deposition, culminating in localized fibrosis [4,14,[21], [22], [23], [24]]. Similar processes have been described in CPTE, where persistent vascular obstruction and local inflammation may promote fibrotic remodeling of adjacent parenchyma. Hirosako et al. also reported that alveolar hemorrhage may play a role in fibrosis development, where oxidative damage from free hemoglobin along with histological findings such as hemosiderin-laden macrophages and cholesterol granulomas, suggests that alveolar injury may contribute significantly to fibrotic remodeling [17,[25], [26], [27], [28], [29], [30], [31]]. Recent advances in diagnostic imaging and understanding of molecular mechanisms have highlighted oxidative stress as a likely key driver of fibrogenesis in these cases. This suggests that antioxidant therapies could represent a promising future strategy, especially if targeted directly to the affected lung parenchyma. In advanced stages of fibrosis, additional therapeutic approaches may include embolization of systemic collateral vessels—primarily to reduce the risk of complications such as alveolar hemorrhage—and, in select cases, pneumonectomy. Although antifibrotic agents (as pirfenidone and nintedanib), have been approved for the treatment of progressive fibrotic lung diseases, their role in UPF remains controversial. This is due to the typically non-progressive nature of the disease and the rarity of UPF, which has precluded large-scale clinical trials assessing the efficacy of these therapies in this specific context [32,33]. In this context, radiology plays a crucial role in recognizing and characterizing unilateral fibrotic lung diseases, allowing clear differentiation based on key features as volume loss, perfusion asymmetry, vascular attenuation, and systemic collateralization. Through detailed imaging analysis, it becomes possible to link structural and vascular abnormalities, clarifying the underlying cause of fibrosis. To conclude, our case series contributes to the literature by describing 3 distinct vascular causes of UPF within a single paper, illustrating the etiological heterogeneity of this condition. While UAPA (unilateral pulmonary agenesis) and CPTE are established, albeit uncommon, causes, the case of UPF secondary to isolated hypoplasia of branches of the pulmonary artery offers a more granular example of a congenital anomaly leading to localized fibrosis. This spectrum - from major vessel agenesis (UAPA) to acquired occlusion (CPTE) and to segmental hypoplasia - highlights the need for radiologic evaluation to identify vascular abnormalities and if necessary to perform detailed vascular imaging, as Computed Tomography Angiography (CTA), in the workup of unexplained UPF.

## Ethics approval

The study was approved by the Ethics Committee of our University hospital. The study was performed in accordance with the ethical standards laid down in the 1964 Declaration of Helsinki.

## Availability of data and materials

Written informed consent was approved and signed from all the 3 patients described in this case series. Appropriate consents, permissions and releases were obtained and retained by the author and are available from the corresponding author on reasonable request.

## Patient consent

The study was performed in accordance with the ethical standards laid down in the 1964 Declaration of Helsinki. Written informed consent was approved and signed from all the 3 patients described in this case series. Appropriate consents, permissions and releases were obtained and retained by the author and are available from the corresponding author on reasonable request.
